# Water Spectral Patterns Reveals Similarities and Differences in Rice Germination and Induced Degenerated Callus Development

**DOI:** 10.3390/plants10091832

**Published:** 2021-09-03

**Authors:** Zoltan Kovacs, Jelena Muncan, Nobuko Ohmido, George Bazar, Roumiana Tsenkova

**Affiliations:** 1 Department of Measurements and Process Control, Institute of Food Science and Technology, Hungarian University of Agriculture and Life Sciences, Somlói út 14-16, 1118 Budapest, Hungary; 2 Biomeasurement Technology Laboratory, Graduate School of Agricultural Science, Kobe University, 1-1 Rokkodai, Nada-ku, Kobe 657-8501, Hyogo, Japan; jmuncan@people.kobe-u.ac.jp; 3 Department of Human Environmental Science, Graduate School of Human Development and Environment, Kobe University, 1-1 Rokkodai, Nada-ku, Kobe 657-8501, Hyogo, Japan; ohmido@kobe-u.ac.jp; 4 ADEXGO Ltd., Lapostelki u. 13, 8230 Balatonfüred, Hungary; bazar@agrilab.hu

**Keywords:** rice, callus proliferation, in vivo monitoring, cell development, near infrared spectroscopy, aquaphotomics

## Abstract

In vivo monitoring of rice (*Oryza sativa* L.) seed germination and seedling growth under general conditions in closed Petri dishes containing agar base medium at room temperature (temperature = 24.5 ± 1 °C, relative humidity = 76 ± 7% (average ± standard deviation)), and induced degenerated callus formation with plant growth regulator, were performed using short-wavelength near-infrared spectroscopy and aquaphotomics over A period of 26 days. The results of spectral analysis suggest changes in water absorbances due to the production of common metabolites, as well as increases in biomass and the sizes of the samples. Quantitative models built to predict the day of the development provided better accuracy for rice seedlings growth compared to callus formation. Eight common water bands were identified as presenting prominent changes in the absorbance pattern. The water matrix of only rice seedlings showed three developmental stages: firstly expressing a predominantly weakly hydrogen-bonded state, then a more strongly hydrogen-bonded state, and then, again, a weakly hydrogen-bonded state at the end. In rice callus induction and proliferation, no similar change in water absorbance pattern was observed. The presented findings indicate the potential of aquaphotomics for the in vivo detection of degeneration in cell development.

## 1. Introduction

All living organs are made of cells, which are small membrane-bound compartments filled with a concentrated aqueous solution of metabolic components. Investigations of cell development are of importance not only for a better understanding of cell biology, but for practical applications as well. A number of biochemical and morphogenetic studies have been carried out to explore the basics of growth and development in plant cells. These studies have mainly been concerned with the detection of changes in concentrations of biochemical components, nucleic acid content, protein synthesis and/or other biomolecules [1,2,3,4]. Despite making valuable contributions, the current state of the art technologies for plant cell development research rely on destructive methods of investigation, and laboratory analyses are performed in vitro on single isolated compounds. The use of non-destructive spectroscopy methods for in vivo investigation, without any disruption of the system dynamics, could lead to a better understanding of the complexity of the cell development process. Apart from bringing new insights to developmental biology, this provides a new non-invasive tool for growth and development monitoring and control. 

In this regard, near-infrared (NIR) spectroscopy is a powerful, non-destructive and rapid technique, which has been used for decades in the agricultural industry to monitor the composition of plant tissues [5] and can proffer a very useful new modality for studying plant cell development as well. Visible–NIR spectroscopy applies the visible and near-infrared light (400–2500 nm) of the electromagnetic spectrum as a probe to measure various physical and chemical parameters of the sample of interest in a completely nondestructive way [6]. It is a quick and highly accurate analytical technique developed to measure the qualitative and quantitative parameters of various products [7]. Although direct association with certain components is difficult because of the overlapping overtones in this region, the technique is beneficial for monitoring and functional studies. Combined with chemometrics, it gives the option for qualitative and quantitative analysis [8]. The excellent performance of NIR spectroscopy in various analytical tasks, such as the quantification of a wide variety of chemicals in plant tissues, for example proteins, lipids, carbohydrates, moisture and amino acids, among many others, has been extensively demonstrated [9,10,11,12,13,14]. 

Short-wave NIR (SWNIR) spectroscopy utilizes light of the 700–1000 nm wavelength to probe the samples, which is very suitable for in vivo studies since these frequencies offer a better penetration depth (up to 10 mm) [15]. This technique has been widely used in various studies for different quantification purposes, such as the quality evaluation of fruits and vegetables [16,17,18,19], or for the estimation of internal quality [20,21,22,23]. In addition, SWNIR spectroscopy has been proven to be an excellent tool for the estimation of various quality parameters in seeds and grain, such as the identification and discrimination of damage in kernels due to heat and frost [24], while numerous recent studies have achieved excellent results in the assessment of seed viability or vitality with this tool [25,26,27,28,29,30]. Studies showed the successful application of visible (Vis) and NIR spectroscopy for the non-destructive and rapid determination of the moisture contents of rice grains [31,32]. NIR spectroscopy also proved to be applicable for the prediction of protein content in rice samples, which shows the applicability of the technique in supporting the digital phenotyping of rice [33]. Vis/NIR-based hyperspectral spectroscopy has been found useful for non-destructive determination of several grain quality properties and phenotyping of rice [34]. NIR spectroscopy combined with different chemometric classification techniques could be also applied for the rapid and accurate authentication of rice [35].

Aquaphotomics [8], as an advanced scientific field, has furthered the application of near-infrared spectroscopy for the exploration of aqueous and biological systems through the rapid and comprehensive analysis of water–light interaction [8]. In this framework, water absorbance bands and water absorbance patterns provide information about the state of the analyzed system, shaped by all of its components and environmental influences, which all leave a characteristic imprint on its intrinsic water matrix. This is the basis of the so-called aquaphotomics water-mirror approach [8,36,37]. NIR spectroscopy and aquaphotomics have been successfully applied in diverse life science applications [37]. In microbiology, for instance, aquaphotomics has allowed the classification and identification of different bacteria cells, as well as a better understanding of their functionality [38,39,40], while in plant biology aquaphotomics was successful in the early diagnosis of virus infection [41], as well as the detection and better understanding of how plants cope with abiotic stress on a molecular level [42,43]. All this was obtained from the water signal, entirely in vivo, without any influence on the physiology of the samples, and in a noninvasive manner [44]. These studies have deepened the understanding of developments made during the living process, with regards to changes in the hydrogen bonding of the water in the respective living systems, thus providing a rationale to further aquaphotomics inquiries to other systems and other processes, such as cell development, germination and plant growth. 

To the authors’ knowledge, these techniques have not yet been applied to describe the water structural changes occurring in a living system during plant cell development in order to discover the relationship between water species and their functionality. With this research, we aimed to examine the process of development during rice germination and seedling growth and callus proliferation by in vivo monitoring using SWNIR spectroscopy and aquaphotomics. The comparison of normal seed growth and callus formation would provide insights into the differences between the two when described in terms of water molecular changes. Therefore, the objective of this research was to investigate the ability of aquaphotomics SWNIR transmittance spectroscopy to detect and describe normal and abnormal cell development, using rice seeds as an experimental material. 

## 2. Results and Discussion

**Figure 1:**
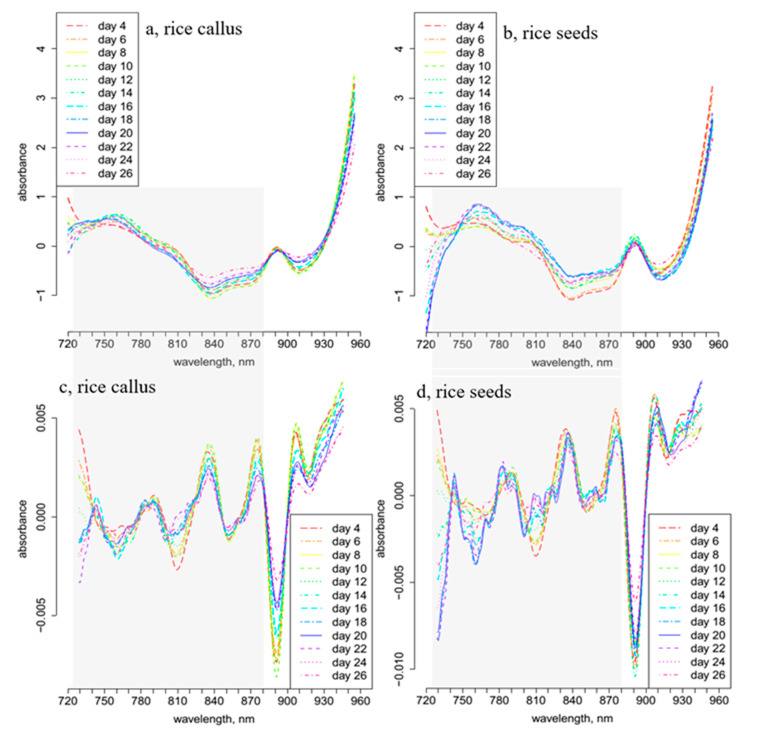
Smoothed, standard normal variate (SNV)-transformed and linear detrend-corrected daily average transmittance spectra of (**a**) 28 callus samples, (**b**) 28 rice seedlings; 2nd derivative of the daily average spectra of (**c**) 28 callus samples and (**d**) rice seedlings.

The daily average spectra and 2nd derivative spectra of callus and rice seeds were calculated and plotted (Figure 1) to examine the spectral changes that occurred during the 26 days of seedling growth, and to find the wavelength ranges showing the largest variation. A relatively sharp and dominant peak was observed at 890 nm in the spectra of both callus and rice seed samples (Figure 1a,b). This region is known as the 3rd overtone of CH and CH_2_ bonds [45,46]. Since both cultures were grown in carbohydrate-rich media, this band at 890 nm can be attributed to sugars, i.e., it has no relevance to the samples investigated. 

Pronounced changes can be observed in the regions between 720 and 780 nm and 800 and 870 nm, corresponding to the 3rd overtone of the OH stretching and OH combination region of water, respectively [47]. The second derivative spectra of rice seedlings (Figure 1d) exhibited more downward peaks in the regions of 720–780 nm and 800–870 nm, in contrast to those of the callus samples (Figure 1c).

Preliminary principal component analysis (PCA) analysis was used as the next step of evaluation to discover the multidimensional patterns of the spectral data and to identify outliers, as explained in detail in the Materials and Methods section. The outliers found with PCA—approximately 30% of the original number of spectra—were eliminated, and quantitative models were built using Partial Least Squares Regression (PLSR) to examine the relationship between the number of days of growth and the spectral data. The regression models were validated using one-seed-out validation, as described in more detail in the Materials and Methods section. The PLSR results can also provide information about the wavelength regions that are most important in the regression model, thus revealing which absorbance bands are most important with respect to growing time.

**Figure 2:**
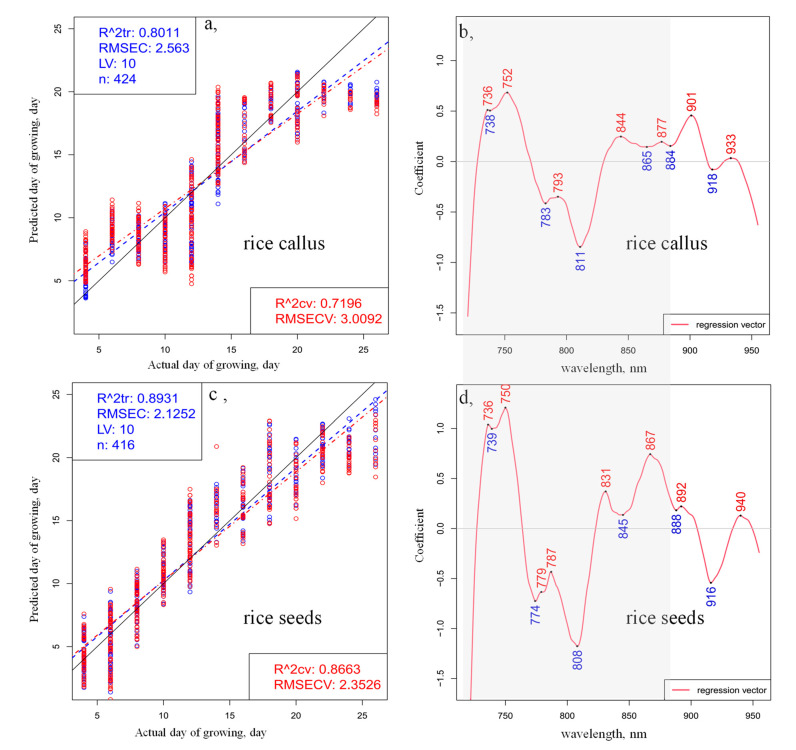
PLSR analysis using time as a dependent variable (the day of development) performed on the preprocessed (smoothing, SNV and linear detrend) transmittance spectra in the spectral range between 720 and 955 nm of (**a**) 28 callus samples and (**c**) 28 rice seedlings; regression vector of the PLSR models for (**b**) callus samples and (**d**) rice seedlings (blue circles indicate the calibration and red circles the cross-validation data points; blue dashed lines indicate the calibration Y-fit and red dashed lines the cross-validation Y-fit, while black continuous lines show the Y-fit of ideal prediction).

The results of the PLSR modeling, performed using time as a dependent variable and smoothed, standard normal variate (SNV)-transformed and linear detrend-corrected spectra in the spectral range between 720 and 955 nm, are shown in Figure 2. 

The fitting and the quality parameters of the regression models showed better fitting in the case of rice seedlings (Figure 2c) compared to the callus (Figure 2a): the coefficients of determination (R2) were found to be 0.89 and 0.80, respectively. The models present relatively high R2 values and low prediction errors, confirming that spectral variation can be used to determine the general change in growth, despite the large within-days variation of the callus and rice seedlings. The Y-fit plots (Figure 2a,c), which compare the actual day of growth versus the predicted day of growth of callus and rice, respectively, both show deviations of linearity, but in the case of the rice callus model, a sharp break in linearity is especially evident around the 14th and 22nd days of monitoring. The regression vectors of the PLSR models (Figure 2b,d) for callus and rice seedling growth appear to be similar. Some differences could be observed in the 770–780 nm, 830–840 nm and 860–870 nm regions, where the regression vector of the PLSR model of the rice samples illustrated more dominant peaks, implying the greater intensity of changes in the somatic cells compared to the callus cells during the monitored period of germination and seedling growth. It is also interesting to note that the regression coefficients in the regression vector of the PLSR model built for somatic rice seed development were nearly double the values of those for callus rice, which speaks of the higher influence of all variables in the former model. This implies that the water matrix in the seeds was more susceptible to changes as time progressed. Based on the above detailed results of the PCA and PLSR analyses of the entire wavelength region (720–955 nm), the wavelength region between 730 and 870 nm was subjected to more detailed evaluation.

The range above 870 nm was removed to avoid the possible negative influence of the bands found to be related to the growing medium around 890 nm. The wavelength interval below 730 nm did not show a clear tendency in the transformed average spectra, but it showed an unexpectedly high weight in the PLSR models, which can be a sign of overfitting caused by the noisy region of the spectrum; therefore, the truncation was further extended. This truncation of the ranges containing non-relevant signals offers the opportunity to better explore the wavelength range found to be important to the changes related only to cell development. 

**Figure 3:**
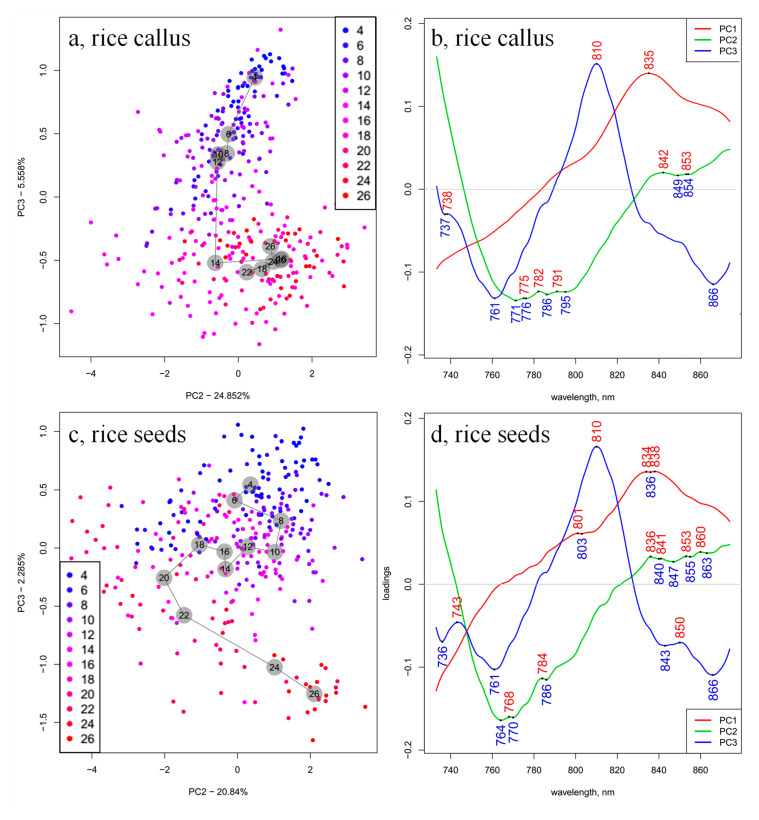
PCA scores (PC1 and PC3, or PC2 and PC3) of (**a**) 28 callus samples and (**c**) 28 rice seedlings, and the loadings of (**b**) 28 callus samples and (**d**) 28 rice seedlings calculated on the smoothed, SNV- and linear detrend-corrected transmittance spectra in the spectral range between 730 and 870 nm (numbers in the gray circles show the group center for the different days of growing).

Hence, PCA evaluation was performed again in the spectral range 730 to 870 nm, on the smoothed and SNV- and linear detrend-corrected spectra. The PCA models of callus rice and rice seedlings samples show about 90% and 95% of the spectral variation in the first three principal components (PCs), respectively (Figure 3a,c). The score plot of the rice seedlings presents higher and more consistent variation, explained by the PC1, but PC2 and PC3 presented more prominent differences for callus rice and rice seedling samples; therefore, the data are not shown for PC1 scores, but they are presented in Figure 3a,c for PC2 and PC3. 

The loading plots of the PCA models (Figure 3b,d) demonstrate the contributions of the different wavelengths in the first three PCs. The first loading, which explained the highest variation in both cases (59.95% in callus samples and 75.71% in seedlings samples), showed one common dominant feature at 835 nm, which could be attributed to water [48]. The shape of the first loading vector closely resembles the shape of raw spectral profiles, indicating that the majority of variations were due to the baseline shift. In the NIR wavelength range, the baseline of the sample absorption spectra was strongly influenced by changes in the sample temperature [49,50,51], which, in the second overtone region of water, manifested as a vertical shift [52]. From the corresponding score plots, the large spreading of scores could be seen along the direction of PC1. In this region (830–840 nm), both water and carbohydrates display absorption [6], but considering that water is a major component of cells (~70%) and hence the dominant absorber [23,53], it is more likely that this feature arises from the water in the samples. Miyamoto and Kitano found that wavelengths close to this particular band (835 nm) are highly correlated with sample temperature [54]. Similarly, the band at 794 nm, which in our case could be detected as a subtle shoulder in the PC1 loading vectors, was also found to be highly correlated with sample temperature [54]. Numerous studies reported similar bands to be absorbance bands of water, which are most affected by temperature: 838 nm [55]; 841 nm [56]; 796 nm and 836 nm [57], and 837.5 nm [58].

Since both these features are present in the loadings of the first PC, it can be concluded that the variation in scores along the PC1 axis was due to the differences in the temperature’s effects on the water matrix of the samples during spectral measurements, which were highly possible given that, in the experimental setup, the seeds were in close contact with the light-emitting probe, and it was not possible to ensure same temperature conditions. Furthermore, the spectra were acquired consecutively, and in this case, the spectral changes resembling temperature-induced spectral patterns are due to the repeated light exposure [59]. It is interesting to note here that this factor, in the case of the callus samples, explained a significantly smaller portion of the total variation (only ~60% compared to ~76% in the seedlings). This difference in variation in the water matrix between the two types of samples may indicate that the water in the rice seeds, in contrast to the callus, was more responsive to temperature perturbation. The differences in the response of the water matrix of the samples to temperature variations indicate differences in the hydrogen bond network as a result of differences in the biochemical composition [60,61,62,63].

The loading vectors of PC2 indicate differences between the two cultures in the regions 750 to 800 nm and 830 to 860 nm. The first region was previously found to be important for the differentiation of types of bacteria based on the metabolites they produce [64], as well as other microorganisms [65]. It may also be the case here that this region of the spectra captures the information related to either differences in metabolites produced by different cell types (callus versus somatic), or simply their different structures.

The second mentioned region is probably related to the increase in the path length of the samples, related to the change in size due to the growth, which is captured during measurements. Miyamoto and Kawano found that, in this region, the absorbance at a wavelength of 840 nm became stronger with the increase in path length [54]. This explanation seems probable, since the measurements were performed during a period of time when, naturally, due to the growth, the sample size changes, and additionally, these differences may have occurred due to different positions of seeds during measurements, resulting in different optical path lengths. 

From the score plot of callus samples presented in Figure 3a, a certain trend could be observed along PC3 with respect to the time of monitoring. In rice seedlings (Figure 3c), during the first days of monitoring, the scores were be located in the positive part of PC3, but moved to the negative part as the time progressed. Although there was a fluctuation in scores on days 16 and 18, there was agreement with the deviations from the linear relationship with time seen in the previous regression analysis. The major feature of the PC3 loading vector was a positive peak at 810 nm. In the case of callus samples, scores at the beginning of monitoring were located in the positive part of PC3, and moved towards zero with time progression, but this came to a halt after the 14th day, with only slight variations afterwards. This also agreed with what was observed when the relationship with time was modeled in the regression analysis. The major feature of the PC3 loading vector in this case was also a positive peak located at 810 nm. This result means that, for both rice seedlings and callus samples, the absorbance at 810 nm decreases with time, with the exception that in callus, around day 14, this trend stops. 

The absorbance band at 810 nm was connected in several research studies with the oxidative metabolism and the state of cytochrome C oxidase (unit IV of the mitochondrial respiratory chain) in various cell types, as well as cell proliferation [66,67,68,69,70]. Since the peak at 810 nm is the common, dominant feature of both PC3 loading vectors, PC3 captures the variance related to the cell mass density during the monitoring period, i.e. the proliferation of cells. 

The differences in the patterns of scores between somatic and callus suggest differences in the growth rate and overall development of the two types of cells; in particular, in the case of callus cells, the fact that there was no change in the scores after 14 days suggests that the cells stopped proliferating. Differences in the PC3 loading vectors for callus and rice seedlings could be observed in the regions 736–738 nm and 830–870 nm. Both regions have previously been found to be important for the prediction of rotting degree [21,22]. In the case of our samples, particular care was taken in the sterilization and isolation of cultures to prevent contamination; however, browning did occur in some of the samples. The fact that the loading vectors between seeds and callus differ in this might be an indication that after day 14, the callus cells stopped growing, and were actually dying (rotting). In both spectral regions, there were vibrational bands of water: the first one could be related to the third overtone of OH stretching vibration (3ν_1_ + ν_3_), while the second one to the second overtone of the combination of stretching and bending vibration (2ν_1_ + ν_2_ + ν_3_) [71,72]. In these two regions, numerous specific water molecular species can absorb: at ~738 nm, hydroxyl ion H_3_O_2_- (3rd overtone) [73]; at 829, 837, 841, 854 862 and 869 nm, various forms of small protonated clusters (+H(H_2_O), +H(H_2_O)_2_), +H(H_2_O)_4_, +H(H_2_O)_6_, [74,75,76,77]; while at around 848, 856 and 871 nm, different forms of superoxide hydrates (-O_2_(H_2_O), -O_2_(H_2_O)_2_, -O_2_(H_2_O)_4_) [78]. The protonated species are considered to be of particular importance for biomolecular reactions in biological systems, while the superoxide ion O_2_- is one of the most important diatomic anions in nature, with a central role in physiological processes, such as aging and inflammation.

In summary, what the PCA results revealed is that the first two PCs capture variation due to the optical path length, i.e., differences in the physical characteristics of the samples. In addition to differences in path length, which are related to the growth of samples, the second PC might also be related to the metabolic compounds produced by cells. The third PC is related to the time progression and the increase in the number of cells, i.e., proliferation and possible rotting of the seed coat tissue. 

**Figure 4:**
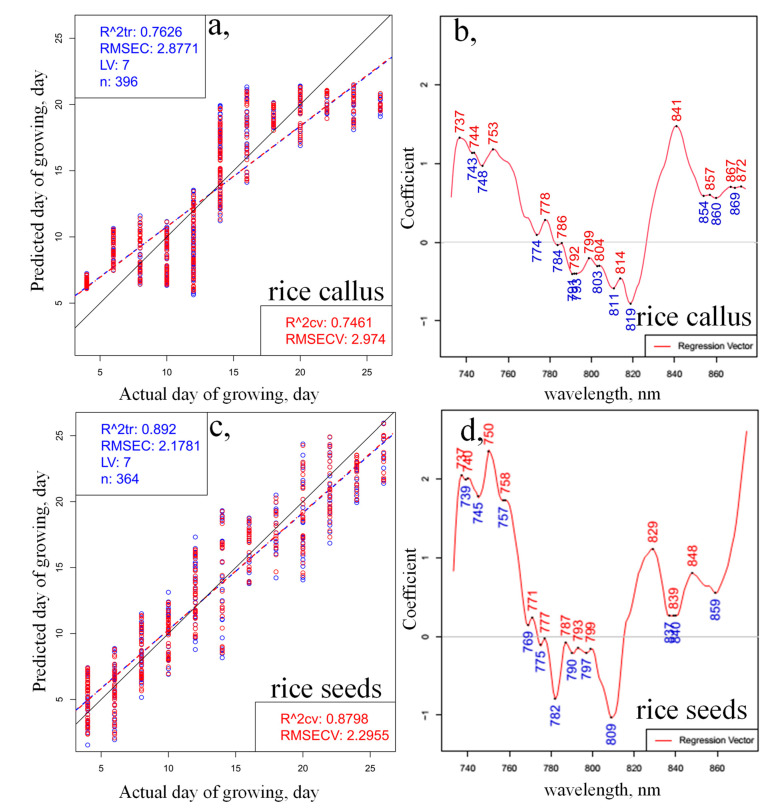
PLSR on the day of growing using the smoothed (calculated with Savitzky-Golay filter using 2nd order polynomial and 31 points), SNV- and linear detrend-corrected transmittance spectra in the spectral range between 730 and 870 nm of (**a**) 28 callus rice seeds and (**c**) 28 somatic rice seeds; regression vector of the PLSR models (**b**) for callus rice seeds and (**d**) for somatic rice seeds (blue circles indicate the calibration and red circles the cross-validation data points; blue dashed lines indicate the calibration Y-fit and red dashed lines the cross-validation Y-fit, while black continuous lines represent the Y-fit of ideal prediction).

Next, the same truncated spectral region (730–870 nm) was used to build PLSR models to regress on the growing time based on the spectral data. The model fitting and quality parameters of the models, similarly to the evaluation performed on the wider spectral range, resulted in better fitting for the rice seedlings (Figure 4a) compared to the results for callus (Figure 4c). The coefficients of determination in the model validation step, using one-seed-out cross-validation, were 0.7461 and 0.8798 for the prediction of callus and somatic rice seeds, respectively, which provided slightly better fitting compared to the modeling over a wider spectral range. The regression vectors of the PLSR models (Figure 4b,d) presented the largest differences between the models of callus and somatic rice growth, around the 730–750 nm, 760–800 nm and 830–870 nm regions, similarly to the results of PCA. It is interesting to note that in both models (Figure 4a,c), the Y-fit plots, showing agreement between the actual and predicted days of growing, display some breaking of the linear trend after two weeks of monitoring, and in the last two days of monitoring. 

In the results of both the PCA and PLSR analyses, high deviations in the scores were observed. As suggested earlier, this was probably a result of the different temperatures of the samples during measurement, differences in the path length due to the geometry or shape, as well as the size of the samples, which was not only different initially, but was also constantly changing due to the growth over the monitoring time. This last phenomenon was observed visually as well.

**Figure 5:**
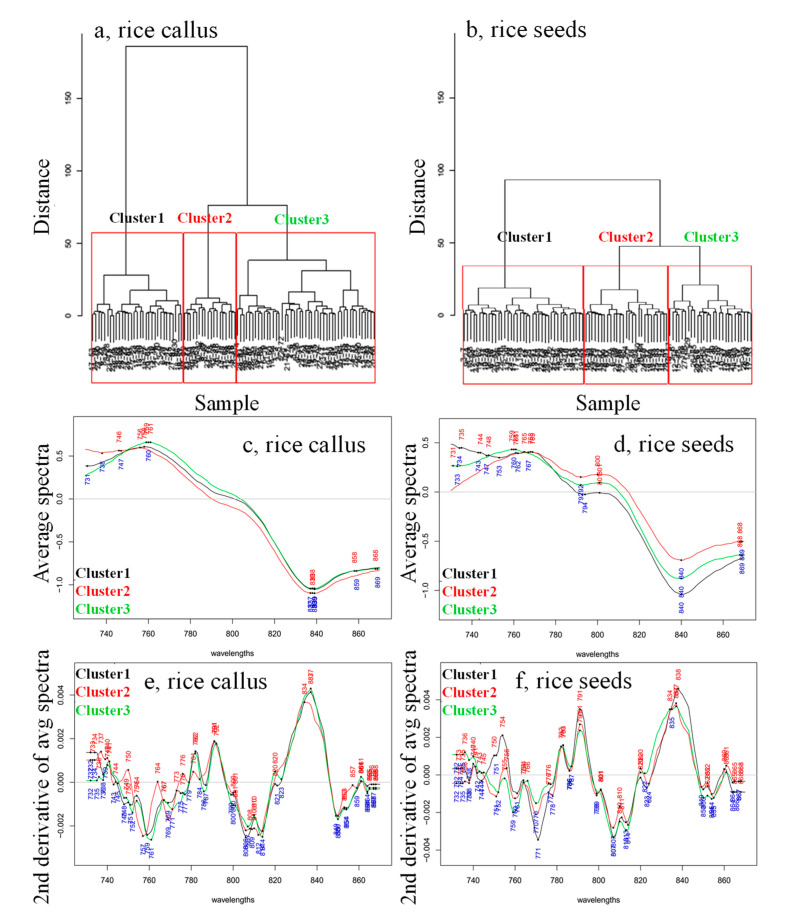
Hierarchical cluster analyses dendrogram calculated on the smoothed (calculated with Savitzky–Golay filter using 2nd order polynomial and 31 points), SNV- and linear detrend-corrected transmittance spectra acquired on the 10th day of growing of (**a**) the 28 callus rice seeds and (**b**) the 28 somatic rice seeds. Average spectra calculated for each of the three clusters found via HCA analysis for the (**c**) callus rice seeds, (**d**) the somatic rice seeds and the 2nd derivative transformation of the averaged spectra for (**e**) callus rice seeds and (**f**) somatic rice seeds.

For this reason, both the callus’ and rice seeds’ spectra were subjected to hierarchical cluster analyses (HCA) to group the data based on their similarities. This analysis was performed on spectra collected for each day separately, which resulted in the repeated identification of three clusters for both somatic and callus samples (Figure 5 is an example of three clusters detected in the spectra collected on the 10th day of monitoring). This result shows that at least three clusters existed in both callus and rice seedlings samples, which showed differences (physically and chemically—different growing speed, different size, shape, etc.) during the monitoring period. 

For each of the identified clusters, the average spectra and their second derivatives were calculated in order to explore the differences in the clusters found within the callus and rice seeds (Figure 5c–f). The average spectra showed subtle differences in the baseline for both callus and rice seeds, and the spectral region from 730 to 800 nm brought out differences between the clusters of biological replicates, which seemed especially different for rice seeds. To better extract the absorbance bands related to these differences, second derivative transformation canceled out the differences in the baseline and improved the resolution of the overlapped bands (Figure 5e,f).

The second derivative spectra showed interesting features—despite differences in cultures, callus and seeds, as well as the differences in biological replicates between different clusters, for the most part, the spectra feature the same absorbance bands. The shapes of the band differed slightly, or showed very small shifts. For instance, the one at 834 nm showed such differences, but mainly, the differences between the somatic rice seeds and callus rice seeds, and between the clusters within the same rice cultures, regard differences in intensity at almost the same absorbance bands. These bands were identified as located at 734–736 nm, 738–740 nm, 748–750 nm, 754–756 nm, 759–761 nm, 764–766 nm, 770–773 nm, 776–778 nm, 782–783 nm, 784–787 nm and 791–792 nm. The intensity and shape of the bands were different for different clusters, and also between the rice seeds and the callus in general.

**Figure 6:**
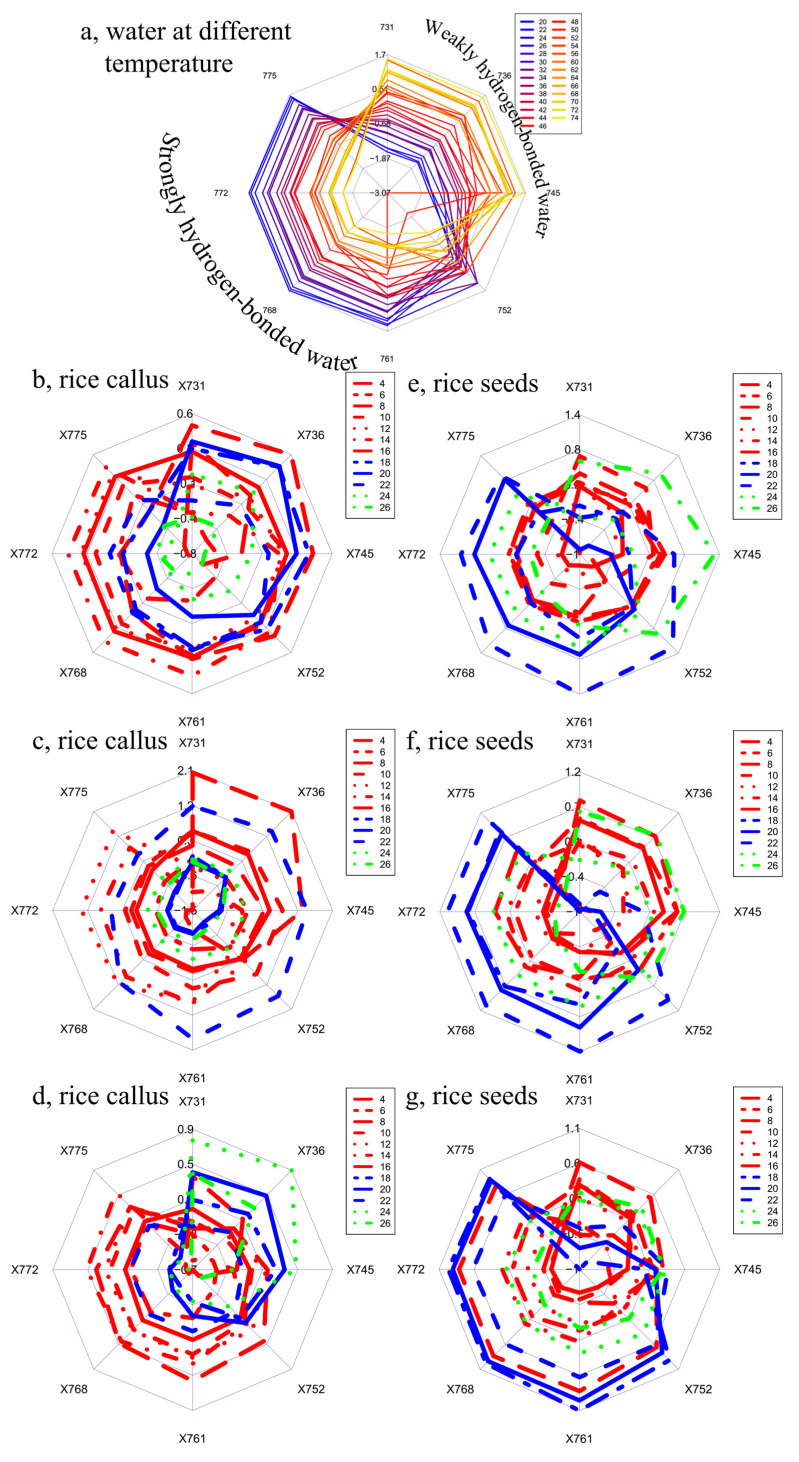
(**a**) Aquagrams of pure water in the 3rd overtone region, at different temperatures, showing fractions of weakly and strongly hydrogen-bonded water, respectively. The aquagram and water fractions are presented for the easier interpretation of the experimental findings, and the numbers in the legend present temperature in °C. Plots (**b**–**g**) present the aquagrams calculated for selected wavelengths, showing the changes during cell development in the 3rd overtone region of water: (**b**–**d**) for the three clusters of callus and (**e**–**g**), for the three clusters of rice seedlings.

Next, the spectra of the three clusters were used separately to calculate aquagrams using the important wavelengths found in the third overtone region of water (720–780 nm) during the qualitative and quantitative evaluation of the spectral data, i.e., the evaluation of the raw and second derivative spectra, and PCA and PLSR evaluation (this region excludes the wavelengths that contained information about the physical differences or temperature). Thus, six aquagrams were calculated: three for the three callus rice clusters (Figure 6b–d) and three for the three rice seedlings clusters (Figure 6e–g), respectively. The aquagrams showed changes in water molecular structure during the observation period of cell development, represented by selected water absorbance bands.

While it is difficult to provide exact assignments of particular water species in the region of the third overtone of water, some general features are well-known. The maximum absorption peak in this region for water vapor is located at 723 nm, while for liquid water near boiling point it is located at 740 nm, for liquid water near freezing point it is at 770 nm, and for ice it is at 800 nm [79]. This means that water species absorbing in the region around 740 nm could be considered to have weaker hydrogen bonds compared to those absorbing around 770 nm, which would resemble more ice-like structures. In order to confirm which species could be considered weakly, and which more strongly, hydrogen-bonded, one more aquagram was calculated showing the changes in liquid water across the wide range of temperatures described by the spectral pattern of changes at the same absorbance bands found to be important for the study of rice development. This aquagram is presented at the top of Figure 6 (Figure 6a), and shows clearly that the water species absorbing at 731, 736 and 745 nm, which are more represented in hot water, could be considered weakly hydrogen bonded compared to those absorbing at 752, 761, 768, 772 and 775 nm, mainly present in cold water, and are characterized by stronger hydrogen bonding. With this new insight, the interpretation of changes in the water matrix of rice seeds and callus could be explained in terms of changes in hydrogen bonding.

The spectral changes in the three clusters of rice seeds presented by the aquagrams (Figure 6e–g) showed a clear tendency as a function of the growing time. A clear, consistent pattern of changes was present in all three aquagrams, indicating common and consistent changes in water molecular structure during the monitoring period, regardless of the differences between the clusters of seeds. This suggests that during development, the water in the somatic cells was probably being affected in a similar way, from the beginning of monitoring up to the 16th day. The water was predominantly in a less hydrogen-bonded state compared to in the period of the 16th day to the 20th, when it changed to a more hydrogen-bonded state, before finally changing back to the weakly hydrogen bonded state during the last days of monitoring. The differences in the state of water from the 4th to the 16th and the 16th to the 20th day, and then beyond, indicate that three different stages of water molecular structure could be identified during the monitoring period of rice seeds. This agrees with the results of PLSR analysis, which showed a deviation in the linear trend in the same periods. The aquagrams of callus (Figure 6b–d) do not depict a clear tendency of the spectral changes in relation to the time of growing. These results are consistent with what was observed in the PCA analysis, where a clear time trend was observed for seedlings, but not for callus samples. These phenomena suggest that the structural changes of water seem more random during the cell development of callus, probably due to the fact that they are undifferentiated, totipotent, unorganized cell masses, which can develop in different directions (i.e., undergo somatic embryogenesis, or not) [3,80,81].

Our findings are also in agreement with several near-infrared imaging studies of embryonic development in higher organisms, which have repeatedly shown that this process is characterized by changes in the proportion of strongly and weakly hydrogen-bonded fractions of water, which may be a consequence of structural changes in biomolecules [82,83,84]. 

The structural organization of water in a particular state was shown to be of importance in earlier aquaphotomics works related to plant survival. Keeping constant the ratio of water species during dehydration, the drastic reduction of free water molecules and the accumulation of water dimers in the completely desiccated state was shown to be a characteristic of plant resurrection—specifically, in Haberlea rhodopensis, one of the rare plant species that can survive extremely long periods without water. The cold resistance ability of genetically modified soybean cultivars was found to be related to their ability to retain water in their leaves when in the weakly hydrogen bonded state [42]. These previous works, together with the current one, suggest that the state of the water molecular structure in plant organs has a function, especially in survival. While in the current work, we cannot say more about the function of the three identified stages of water structural organization, the representation of changes in the water molecular matrix of callus and rice seeds, presented in aquagrams, clearly shows the existence of differences between degenerate cell and normal cell growth. While the aquagrams of rice seed clearly indicate the three stages of water molecular structure during the growth of rice seeds, there were no such stages in callus development, and no patterns of changes, indicating the absence of normal growth, i.e., the degeneration and dying of the cells. In other words, using aquaphotomics, it was possible to clearly detect the relation of the water matrix and the spectral pattern of normal growth, and to detect abnormality and cell death. The unprecedented novelty of these findings lies in the fact that this was achieved by utilizing only the spectral response of the water molecular matrix of the cells, in a completely non-destructive way. 

## 3. Materials and Methods

### 3.1. Plant Material 

The experiment was conducted using mature seeds of rice (Nipponbare, *Oryza sativa* L.). The seeds were dehusked manually and surface-sterilized with 70% ethanol for 5 min, followed by a 5% commercial solution of sodium hypochlorite (NaOCl_2_), and then rinsed thoroughly three times with sterile distilled water.

### 3.2. Culture Preparation 

Twenty-eight seeds were prepared for each task—rice seedling growth and callus induction. The seeds were placed in closed Petri dishes containing agar base medium and kept at room temperature (temperature = 24.5 ± 1 °C, relative humidity = 76 ± 7% (average ± standard deviation)) in a dark box throughout the experiment for the germination and rice seedling growth of the intact seeds. Callus formation was induced from seed explants after seed sterilization and plating onto in vivo tissue culture medium containing N6 basal salt with 3% sucrose, 2.88 g/L proline, 0.3 g/L casamino acid, 0.1 g/L mioinositol, N6 vitamins, 4 g/L gelrite, and plant growth regulator at 2 mg/L 2,4-D to generate callus cells from the mature embryo of rice. 

### 3.3. Instrumentation and Spectral Measurements

A SAIKA instrument (SAIKA Technological Institute Foundation, Wakayama, Japan) equipped with a silicon photodiode array detector attached to a fiber optic cable with a 2 mm diameter controlled by PureSpect Control software (ver. 1.0., SAIKA Technological Institute Foundation, Wakayama, Japan) was used to monitor the growing of the seeds. The transmittance spectra of the individual seeds were recorded for the entire spectral region (660–960 nm) with 1 nm step (301 data points). The monitoring was performed from the 4th to the 26th day of development, by acquiring spectral every other day (12 time points).

Reference was taken once a day at the beginning of the experiment using blank air. The signal of the detector (dark scan) was also acquired once a day before reference acquisition by turning off the lamp and acquiring the baseline spectrum of the instrument. 

**Figure 7:**
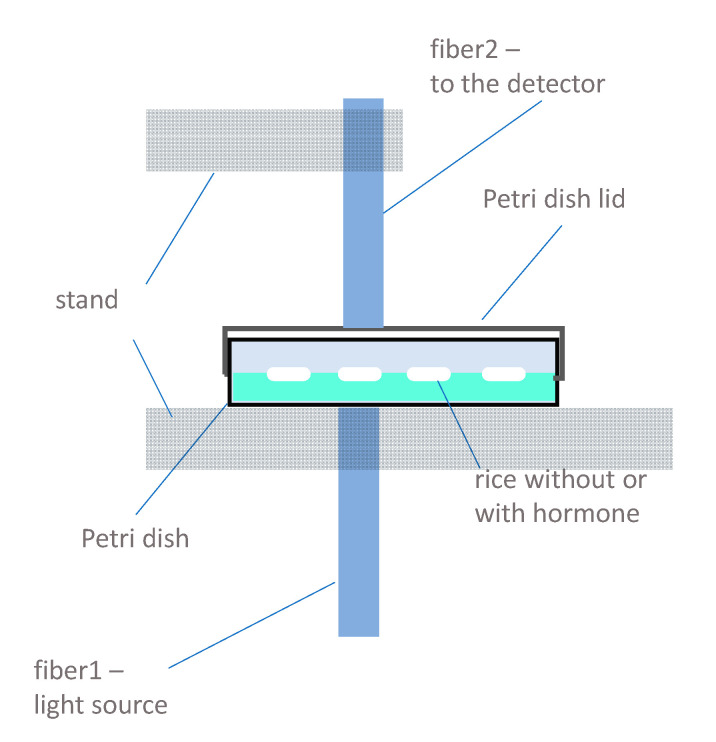
Scheme of the experimental measurement setup.

The fiber probes of the instrument were fixed with a stand with a gap equivalent to the height of the closed Petri dishes. The Petri dishes were placed on the stand and positioned for the individual seeds one by one for the measurement. During the monitoring period, scanning was repeated at the same positions marked on the first day of the experiment, which provided the spectra of the mixture of the original seed and the different developing organs after the germination, as well as for the late phase of seedling growth. The fiber transporting the light was attached from the bottom, while the fiber receiving the transmitted light through the sample was attached to the top of the Petri dishes (Figure 7). Every individual seed was scanned from four different directions, turning the Petri dish three times by 90°, and five consecutive scans were taken, resulting in 20 acquired spectra for each seed at a given time point. The total number of acquired sample spectra was 13,440 (2 types of samples (rice seedling growth and rice callus formation) × 28 seeds × 4 positions × 5 consecutive scans × 12 time points). The Petri dishes were kept closed during the entire experiment to avoid any contamination of the cultures. 

The measured intensity values of the samples, the reference and the dark scan were used to calculate absorbance values, as follows:

$$A_{i}=\frac{R_{i}-D}{S-D}$$

where *A_i_* is the processed absorbance spectrum of the *i*-th sample, *R* is the raw spectrum of that sample, *S* is a reference, and *D* is dark. 

### 3.4. Data Analysis

Preliminary spectra selection was performed based on the visualized absorbance spectra, where the spectra observed to be obvious outliers due to the saturation of detector or other artifacts (e.g., due to the misalignment of fibers) were eliminated. As the next step, the average values of the consecutive scans were calculated at every wavelength. Due to noise and the lower quality at the edges of the collected spectra, the spectral region had to be trimmed to 720–955 nm for further evaluation. Pre-experiments with an empty Petri dish showed that, although the spectra were collected through the polystyrene Petri dish, in this spectral region, polystyrene does not have a characteristic absorption peak. Several combinations of spectral pretreatment (e.g., smooth with different numbers of points combined with multiplicative scatter correction (MSC), standard normal variate transformation (SNV), detrend or derivation) were tested prior to the modeling and finally the following combination was found to be optimal based on the results of the Partial Least Squares Regression (PLSR) models. Smoothing of the spectra was performed using a Savitzky–Golay smoothing filter [85,86] (2nd order polynomial and 31 data points). Due to the differences in size and shape of the seeds, the resulting differences in light path length and light scattering caused baseline and slope effects in the spectra of the different seeds, which had to be corrected. Correction was performed using SNV [87] and linear detrend transformation [88,89]. Every single spectrum was centered and scaled by dividing each data point by the standard deviation of the respective spectrum for the SNV transformation, while linear modeling of the baseline as a function of wavelength and a subsequent subtraction of this function from each spectrum individually was performed for the linear detrend transformation.

After the above-described preprocessing, the spectral data of callus and rice seedling samples were analyzed separately. The daily average of the preprocessed spectra and the 2nd derivative spectra were used to examine the spectral changes, and to determine the wavelength ranges wherein the largest changes occurred during the monitoring period. Principal component analysis (PCA) [90] was used to describe multidimensional patterns of the spectral data and to discover outliers. Outlier detection was performed by executing boxplot analysis on the data from each day for each sample type for the first three PCs, separately. The observations lying beyond the extremes of the whiskers defined as 1.5 times the interquartile range from the median were detected as outliers. PLSR [87] was used to examine the relationship between spectral data and the day of monitoring of callus and rice seeds. The spectra were mean-centered prior to the creation of the models. The PLSR models were evaluated by the coefficient of determination in calibration (R2tr), the root mean squared error of calibration (RMSEC), the coefficient of determination in cross-validation (R2cv) and the root mean squared error of cross-validation (RMSECV). The maximum number of latent variables (LV) used in the regression models was chosen to be equal to the day of growing in order to avoid overfitting. The PLSR models were validated using one-seed-out validation. The dataset was split into training and test sets. The spectral data of 27 seeds were used as the training set, and those of one seed left as the test set. This process of data splitting was repeated 28 times to ensure that the data of all the seeds can be included in the evaluation set once [91]. 

The analyzed seeds showed very high diversity and different growing rates. Hierarchical cluster analyses (HCA) was used to discover any possible groupings among the different seeds based on their naturally different growth rates. The HCA is an unsupervised clustering technique [91] that groups the samples on the basis of distances without taking into account the information about class membership. In this study, cluster analysis was performed via Ward’s method using Euclidean distances [92]. HCA was performed in steps using data separately obtained on each day of the experiment for callus rice and somatic rice seeds, resulting in 12 cluster dendrograms each for both callus rice and somatic rice. Each of the daily cluster dendrograms were grouped into three clusters, and the appearance of the individual seeds in the different clusters were recorded. Finally, each seed was assigned to one of the three clusters based on its most frequent appearance. The purpose of this HCA analysis was to eliminate the high variations in the different growth rates of the seed, making it possible to visualize their water spectral pattern using the preprocessed averaged spectra, 2nd derivative-averaged spectra, and aquagrams.

The above-mentioned methods enabled the identification of wavelengths in the spectra that showed the largest changes in absorbance during the development. Those wavelengths were used to calculate and visualize aquagrams [89,93] in order to present cell growth dynamics as a function of absorbance at particular water matrix coordinates (WAMACs) [8]. An aquagram is a star-chart that displays normalized and averaged absorbance at the selected wavelengths corresponding to specific water absorbance bands [93], and it can be a useful visualizing tool to better observe the dynamics of water structural changes along some perturbation of interest [89]—in the case of this study, how the water structure in the rice seedlings and callus cells changes during the monitoring period. 

The calculation and visualization were performed in the R-project environment using R Studio user interface [94,95].

## 4. Conclusions

Aquaphotomics coupled with SWNIR spectroscopy was applied to monitor the water structural changes during callus formation and the proliferation of plant cells in the germination and seedling growth of rice from the 4th to the 26th day of cell development.

The use of the aquaphotomics concept provided the possibility for the non-invasive, real-time monitoring of the water molecular structure changes of callus and somatic rice cells as integrative spectral markers for screening. Qualitative models revealed similarities in the main water absorbance bands activated in cell development, and differences in the spectral patterns and dynamics between the developmental process of callus and rice seedlings. PCA analysis revealed that absorbances at certain water absorbance bands and spectral regions can be related to the change in size of the samples, as well as to an increase in the number of cells. Partial least squares regression (PLSR) results showed that it was possible to predict the day of the development with better accuracy for rice seedlings growth (R2cv = 0.88, RMSECV = 2.3 day) compared to callus formation (R2cv = 0.75, RMSECV = 2.97 day). Aquagrams were used to visualize the spectral changes occurring at the selected water absorbance bands during cell development. The aquagrams of rice seedling growth clearly showed the direction of spectral changes as a function of the growing time, but nothing similar was observed for callus formation. The results suggest that structural changes in water are more random in non-differentiated callus cells, but show a clear tendency in vegetative cells during cell development. Therefore, the tested seedling growth period could clearly be described by three stages: the 4th to the 16th, the 16th to the 22nd and the 22nd to the 26th days, which mark the transition from water being in the weakly hydrogen-bonded state, to being strongly hydrogen-bonded, and then again weakly hydrogen-bonded. These findings show clearly that, using aquaphotomics, it was possible to detect differences between degenerate cell and normal cell growth. The promising results of SWNIR spectroscopy coupled with aquaphotomics suggest the strong potential of the technique for rice seed authentication and characterization, and beyond that, for detection of abnormalities in growth and development, which may offer excellent feedback for early warning systems. Based on the results of this preliminary investigation, further and more robust studies will be initiated, which will test the possibilities for transfer to in-field applications. Additionally, more in-depth studies with different plant species are planned, with the goal of further inquiring into the role of water’s molecular structure in seed germination, seedling growth and normal development. 

## Data Availability

The data presented in this study are available on request from the corresponding author. The data are not publicly available due to privacy and ethical reasons.
